# Diabetic Retinopathy and Eye Screening: Diabetic Patients Standpoint, Their Practice, and Barriers; A Cross-Sectional Study

**DOI:** 10.3390/jcm11216351

**Published:** 2022-10-27

**Authors:** Naif Mamdouh Alali, Alanuad Albazei, Horia Mohammed Alotaibi, Ahad Massd Almohammadi, Eilaf Khaled Alsirhani, Turki Saleh Alanazi, Badriah Jariad Alshammri, Mohammed Qasem Alqahtani, Moustafa Magliyah, Shaker Alreshidi, Hani B. Albalawi

**Affiliations:** 1 Division of Ophthalmology, Department of Surgery, Faculty of Medicine, University of Tabuk, Tabuk 47512, Saudi Arabia; 2 Medical Education Department, King Khaled Eye Specialized Hospital, Riyadh 11462, Saudi Arabia; 3 Ophthalmology Department, Imam Abdulrahman bun Faisal University, Damman 34212, Saudi Arabia; 4 Pediatric Department, Johns Hopkins Aramco Health Care, Dhahran 34465, Saudi Arabia; 5 Ophthalmology Department, King Fahad Specialist Hospital, Tabuk 47913, Saudi Arabia; 6 Internal Medicine Department, King Salam Armed Forces Hospital, Tabuk 47512, Saudi Arabia; 7 Obstetrics and Gynecology Department, King Salam Armed Forces Hospital, Tabuk 47512, Saudi Arabia; 8 Preventive Medicine Department, King Salam Armed Forces Hospital, Tabuk 47512, Saudi Arabia; 9 Ophthalmology Department, Prince Mohammed Medical City, Sakakah 42421, Saudi Arabia; 10 Ophthalmology Department, Almajmaah University, Almajmaah 15341, Saudi Arabia

**Keywords:** diabetic retinopathy, knowledge, practice, attitude, Tabuk, Saudi Arabia

## Abstract

Diabetes mellites (DM) is one of the most common systemic disorders in Saudi Arabia and worldwide. Diabetic retinopathy (DR) is a potentially blinding ophthalmic consequence of uncontrolled DM. The early detection of DR leads to an earlier intervention, which might be sight-saving. Our aim in this cross-sectional study is to assess patients’ knowledge and practices regarding DR, and to detect the barriers for eye screening and receiving a check-up from an ophthalmologist. The study included 386 diabetic patients. One hundred and thirty-one patients (33.9%) had T1DM and 188 (48.7%) had T2DM. Most of the diabetic patients (73.3%) know that they must have an eye check-up regardless of their blood sugar level. DM was agreed to affect the retina in 80.3% of the patients, 56% of patients agree that DM complications are always symptomatic, and 84.5% know that DM could affect their eyes. The fact that blindness is a complication of diabetic retinopathy was known by 65% of the diabetic patients. A better knowledge was detected among patients older than 50 years of age (54.9%) compared to those aged less than 35 years (40.9%), which was statistically significant (*p* = 0.030). Additionally, 61.2% of diabetic patients who were university graduates had a significantly better knowledge in comparison to 33.3% of illiterate patients (*p* = 0.006). Considering the barriers to not getting one’s eyes screened earlier, a lack of knowledge was reported by 38.3% of the patients, followed by lack of access to eye care (24.4%). In conclusion, there is a remarkable increase in the awareness of DR among the Saudi population. This awareness might lead to an earlier detection and management of DR.

## 1. Introduction

Diabetes mellites (DM) is one of the most common metabolic disorders which affects insulin production or action, such as insulin resistance. It is divided into two types, type 1 DM, in which there is hyperglycemia due to an absolute insulin deficiency, and type 2 DM, which is also characterized by hyperglycemia due to relative deficits in insulin secretion and action [1].

The global prevalence of DM is estimated to be around 8.5% among adults older than 18 years in 2014 [2]. The overall prevalence of DM in Saudi Arabia from 1995–2000 among people who were 30 years or older was 23.7% [3], which is much higher than the worldwide prevalence, indicating that DM is a serious health issue among Saudis.

Diabetic retinopathy (DR) is a retinal consequence of chronic diabetes at the microvascular level. It is one of the most common causes of blindness in diabetic patients. It accounts for about 20% of new blindness incidents among patients who are 45–74 years old [4]. The prevalence of DR among Saudi diabetics is 31% [5]. Types of DR include non-proliferative diabetic retinopathy (NPDR) and proliferative diabetic retinopathy (PDR). The American Academy of Ophthalmology’s recommendations for a follow-up of DR are as follows for normal or mild NPDR: every 12 months, for moderate NPDR with clinically significant macular oedema: every 1 month, for severe NPDR without clinically significant macular oedema: every 2 to 4 months, and for severe NPDR with clinically significant macular edema: every 1 month [6]. Regular follow-ups with the ophthalmologist is key to prevent DR progression and blindness and allows for earlier interventions and the management of DR.

A study from the Southern region of Saudi Arabia showed that one third of diabetic patients who has been followed in a diabetic center had DR. Additionally, the study showed that retinopathy is heavily associated with people who were of an older age, had an earlier-onset or longer duration of DM, a poor control of their blood sugar, the use of insulin in their treatment, and the coexistence of hypertension [7]. The aim of this study was to assess diabetic patients’ knowledge and practices regarding RD, and to detect the barriers for eye screening and check-ups with ophthalmologists among diabetic patients in Tabuk, Saudi Arabia.

## 2. Materials and Methods

This was a cross-sectional study of the knowledge and practices of the diabetic patients of Tabuk, Saudi Arabia regarding DR, as well as the barriers for eye screening and check-ups among the population of Tabuk, Saudi Arabia. It was a hospital-based study at the King Fahad Specialist Hospital Tabuk (KSFH) and King Khaled Hospital (Medical and Surgical wards). The study was conducted by distributing the questionnaires among the patients admitted to the wards or who visited the diabetic centers of the KSFH. The ethical approval was obtained from the University of Tabuk. The research started on 25 June 2019 and the patients who were diagnosed with DM type 1 or type 2 were included. Patients younger than 18 years old were excluded from the study.

Data analysis was performed using statistical software IBM SPSS version 22(SPSS, Inc. Chicago, IL, USA). All statistical analyses were done using the two tailed T-test. A *p* value less than 0.05 was statistically significant. For the awareness items, each correct answer was scored one point and the total summation of the discrete scores of the different items was calculated. A patient with a score less than 60% (seven points) was considered to have a poor awareness, while a good awareness was considered if they had a score of 60% (eight points or more). Descriptive analyses based on the frequency and percent distribution were done for all variables, including the patients’ demographic data, diabetes clinical data, awareness items, and patients’ practice and source of information. Cross tabulation was used to assess the distribution of one’s awareness according to the patients’ personal and medical data and the source of information. The relations were tested using Pearson’s chi-square test.

## 3. Results

The study included 386 diabetic patients who were aged between 18 and 65 years. The average age of the patients was 38.7 ± 12.5 years. Among these patients, 203 were females (52.6%) and 147 (38.1%) were university graduated. As for the patients’ monthly income, it was less than 5000 SR (1330 USD) among 161 (41.7%) of the patients. Smoking was reported among 83 (21.5%) of the patients and 63.5% had other co-morbidities including HTN (40.4%), hyperlipidemia (28.8%), and cardiac diseases (15.8%) (Table 1).

Table 2 shows the DM data and related eye diseases among the study’s patients. One hundred and thirty-one patients (33.9%) had T1DM, and 188 (48.7%) had T2DM, while the DM type was unknown for 67 (17.4%) patients. DM was diagnosed 5–10 years earlier in 129 patients (33.4%), while 145 patients (37.6%) had DM for more than 10 years. Oral hypoglycemic medications were used by 202 patients (52.3%), while 172 (44.6%) were on insulin. Checking the blood sugar level at home was reported as “When I feel unwell” among 119 (30.8%) patients, while 88 (22.8%) checked once daily. Ophthalmic diseases were detected in 184 diabetic patients (47.7%) which included cataracts (16.8%), refractive errors (47.3%), and DR (17.9%). Treatments for their eye diseases were received among 117 (63.6%) of those affected.

**Figure 1:**
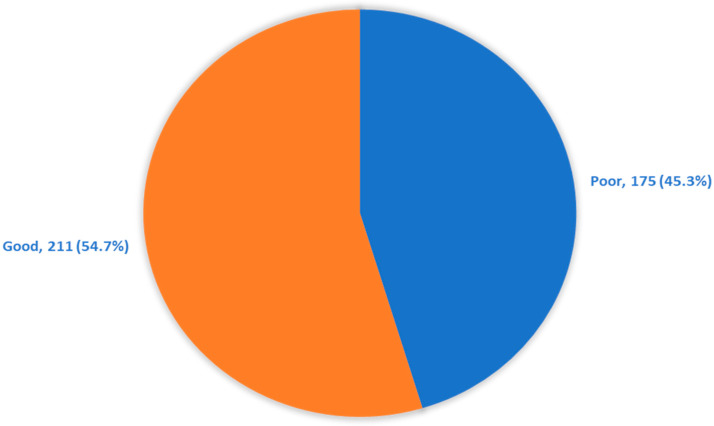
Overall awareness regarding diabetes and diabetic retinopathy among diabetic patients, Tabuk region, Saudi Arabia.

Table 3 illustrates the diabetic patients’ knowledge regarding diabetes and DR. Most of the diabetic patients (73.3%) knew that they must have eye check-ups regardless of their blood sugar level, and 37.8% knew that diabetics should undergo an eye check-up every year. About half of the studied patients (50.8%) did not believe that they needed to have their eyes checked; they believed this was only necessary when they had symptoms. Additionally, 80.3% of the patients agreed that DM can affect the retina, 56% agreed that diabetic complications are always symptomatic, and 84.5% knew that DM can affect their eyes. The fact that blindness is a complication of diabetic retinopathy was known by 65% of the diabetic patients, but only 26.7% knew that there are available treatments for DR. Blood sugar control as a preventive measure for DR was known by 69.9% of patients, and 53.9% knew that they can prevent DR by a good BP control. Additionally, 75.9% of the studied patients knew that they could prevent DR by a regular visit to the ophthalmologists. In total, 211 (54.7%) of the patients had a good knowledge of DM and DR (Figure 1).

**Figure 2:**
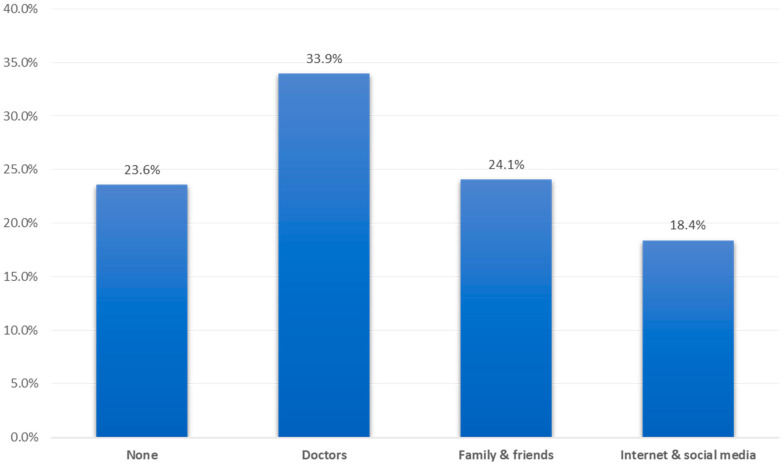
Source of information regarding diabetes and diabetic retinopathy among diabetic patients, Tabuk region, Saudi Arabia.

As for the sources of the patients’ information regarding DM and DR (Figure 2), the most reported source was from their doctors (33.9%), followed by their family and friends (24.1%), and the internet and social media (18.4%), while 23.6% had no source of information.

Table 4 demonstrates diabetic patients’ practices regarding their eye check-ups and screening. Among the studied patients, 282 diabetics (73.1%) had their eyes checked before, including 24.8% who had a check-up in the past 2 years, 28% in the past 1 year, and 32.6% in the past 6 months. Additionally, 25.4% of the patients reported that they do eye screening annually. The most reported causes of receiving an eye screening during the past 12 months were not having received any recommendations from their physicians (18.7%), no having enough time (16.6%), having difficulty in reaching an ophthalmologist (9.8%), being asymptomatic (9.3%), and having a lack of information about diabetic eye diseases (8%). Reasons which made patients undergo their first eye screening included a referral from another physician (43.5%), patient’s awareness about the importance of regular eye examinations (15.55), and a knowledge of the risk of DM on the retina (12.4%). Considering the barriers for not receiving an eye screening earlier, a lack of knowledge was reported by 38.3% of the patients, followed by a lack of access to eye care (24.4%), time limitations (16.6%), and a fear of discovering something bad (11.9%), while the cost was among 8.8%.

Table 5 shows patients’ attitudes regarding the physician’s role in encouraging an eye screening. About half of diabetic patients (57.8%) reported that their primary physicians mentioned their need for a regular follow-up in ophthalmology, 58% reported that primary physicians informed them about the effect of diabetes on their eyes, and 58.3% reported that they were advised to go for an ophthalmic screening. Most of the studied patients (72.3%) thought that diabetics need to have an eye check-up when his/her blood sugar level is well-controlled and 67.9% thought that diabetics need to have an eye check-up when his/her blood sugar level is poorly controlled.

Table 6 shows the determinants of patients’ knowledge level regarding DM and DR among diabetic patients. Good knowledge was detected among 54.9% of patients aged more than 50 years compared to 40.9% of those aged less than 35 years with a detected statically significance (*p* = 0.030). Additionally, 61.2% of university-graduated diabetic patients had a good knowledge in comparison to 33.3% of illiterate patients (*p* = 0.006). A good knowledge was also reported among 69.5% of type 1 diabetic patients compared to 37.3% of those who were unaware of their DM type (*p* = 0.001). Diabetics who had DM for more than 10 years had a significantly better knowledge (68.3%) compared to others who were diabetics for less than 5 years (38.4%) (*p* = 0.001). Additionally, 67.2% of diabetic patients who had their information from their doctors had a good knowledge in comparison to 30.8% of those with no mentioned source (*p* = 0.001). The majority of diabetic patients who were told by their doctors about the effect of DM on their eyes had a better knowledge (67.9%) in comparison to 36.4% of those who were not (*p* = 0.001). Additionally, 62.4% of patients who had undergone an eye check-up had a good knowledge versus 33.7% of those who did not (*p* = 0.001).

## 4. Discussion

DR is one of the major diabetic complications which can lead to blindness. It is asymptomatic in the early course of the disease. Thus, a screening program for diabetic patients has been implemented for the early detection of the disease and thus the treatment. Patients’ awareness is important for following the recommendations and attending annual screening visits [8]. In this study, a hospital-based, cross-sectional study, which documented the KAP patterns of diabetic patients regarding DR in Tabuk city, included 386 diabetic patients and their ages ranged from 18 to 65 years old. Almost 54.7% of patients with diabetes had a good overall awareness of DM and DR (Figure 1).

Among our participants, 84.5% know that DM could affect their eyes. This is consistent with the findings of studies done in high-income countries. According to Schmid et al., 96% of diabetic patients in Australia were aware that DM might cause vision loss [9], and more than 98% of type 2 DM patients in Japan were aware that DM might cause eye impairment [10]. This level of knowledge is probably due to the improved health care and higher awareness of the importance of the prevention and treatment of DM to reduce the risk of its complications among populations.

DR is a leading cause of preventable vision impairment and blindness, and patients’ awareness and compliance are crucial in preventing DR [8]. This study revealed that diabetic patients had a high level of awareness of DR, with more than 65% know that DR can lead to blindness and 80.3% know that DM can harm the retina. Similar findings were reported by AlHargan et al., who found that more than two-thirds of diabetic patients were aware that DR can lead to blindness and 88% were aware that DM can affect the retina [11]. These results might reflect the efforts made by the health care authorities to raise awareness amongst diabetic patients about DR, as well as the availability of more ophthalmologists on the traditional media, social media, and social campaigns to improve the awareness of the population about diabetic retinopathy.

Only 26.7% of the studied patients knew that there are treatments available for DR. This was similar to a study conducted in the Western region of Saudi Arabia, as more than 34% of subjects were not aware that surgery and laser are treatment options for DR [12]. Another study done in India found comparable gaps in treatment awareness and the need for more proactive outreach efforts [13]. This might indicate the need for more efforts from ophthalmologists to increase the awareness of the population about the different treatment options for DR.

About 56% of the participants believed that diabetic complications are always symptomatic, however 50.8% of diabetics thought that they needed to have their eyes screened even if they were asymptomatic. Moreover, 75.9% of the studied population knew that they could prevent diabetic eye disease by a regular visit to the ophthalmologist. Despite the high level of awareness regarding diabetic eye diseases, knowledge about the frequency was not optimal, with only 37.8% knowing that diabetics should undergo annual eye examinations. This is in line with the findings of previous studies. In Riyadh, Saudi Arabia, 28.7% of diabetics believed that they needed a screening once every year [14], while in Jordan only 20.7% thought that an eye examination should be done annually, [15] and 41.9% of people in Turkey believed that an annual eye check is required [16]. The follow-up frequencies need to be elaborated more by ophthalmologists.

Tight glycemic control is known to influence the occurrence of DR [17]. Among patients in this study, 73.3% knew that diabetics need to have regular eye examinations regardless of their blood sugar level. Blood sugar control as a preventive measure for DR was acknowledged by 69.9% of the patients. These results were different from what was found in Riyadh, as only 22.8% of the respondents there believed that a poor glycemic control was an essential factor that worsens diabetic retinopathy [14]. These results highlight the importance of encouraging patients to adhere to the follow-ups scheduled with their ophthalmologists regardless of the blood sugar levels because DR can be asymptomatic in some patients.

As a part of the metabolic syndrome, hypertension is frequently linked to DM. A high blood pressure increases the risk of both the onset and progression of DM. Blood pressure management in diabetic and hypertensive individuals has been proven to delay the onset of DR [18]. Almost 53.9% of the studied population knew that they could halt the progression of diabetic eye disease by a good BP control. These results indicate that more efforts are needed to elaborate the close connection between blood pressure control and the complications of DM.

When addressing the most reported sources of information, physicians were the providers for 33.9% of participants, followed by family and friends for 24.1%, and the internet and social media for 18.4%, while 23.6% had no source of information. These results are consistent with a previous study conducted in Saudi Arabia which revealed that the most reported source was a physician (44.9%), followed by family and the media by 21.6% and 17.9% of people, respectively [19]. These results indicate the need for a more creative means to deliver information about DM and DR in a faster and more feasible way to the patients, such as via the traditional media, social media, and social campaigns.

Our study population reported that the most common cause of not having their eyes screened during the past 12 months was the lack of a recommendation from their physician (18.7%). On the contrary, the most common reason for diabetics to undergo their first eye screening was their physician’s referral (43.5%), while among the barriers for eye screening, a lack of knowledge was the most reported cause in 38.3% of participants. This can be explained by the limited communication between physicians and patients. Nevertheless, Srinivasan et al.’s study in India showed that also a lack of primary physicians’ recommendations was the most common barrier (38.5%) for a periodic eye check-up (20). In the western region of Saudi Arabia, physicians’ referrals were also the most frequent reason for receiving a first eye screening, while a lack of knowledge was the most frequent barrier to not undergoing an eye screening earlier [20]. On the other hand, the most common reason to not comply with regular follow-ups in Riyadh among 47.1% of diabetics was that “they thought it was not important” [14]. These results show that a patient–physician interaction and good communication skills are still the most influential part of a patients’ knowledge about DR and the need for regular follow-ups.

According to 72.3% of the studied patients, diabetics need to have eye examinations even when his/her blood sugar levels are well-controlled. Similar findings were reported in Riyadh as 75.8% believed that they should follow-up with an ophthalmologist even if their blood sugar was under control. [14] These results indicate that most diabetic patients get enough information about the importance of regular follow-ups once they get their eyes checked up by an ophthalmologist.

Significantly more knowledge was detected among patients older than 50 years (*p* = 0.030). On the contrary, in Riyadh, it was found that younger age was associated with more knowledge about DM (*p* < 0.049) [14]. These results might indicate that patients who live in Riyadh, the capital of Saudi Arabia, might have more access to more modern sources of information such as social media and social campaigns, which are commonly accessed by the younger generations. While in other regions, the sources of information might be still more dependent on physicians, who are mostly visited by the elder part of the community.

In this study, 68.3% of diabetics who had diabetes for more than 10 years had significantly more knowledge compared to 38.4% of others who were diabetics for less than 5 years (*p* = 0.001). Similarly, in India, higher positive attitudes were found among patients with a longer duration of the disease (>5 years) [20]. Meanwhile, AL-Yahya et.al found no significant association with the duration of DM and the level of knowledge [14]. These results indicate that more focus is needed on patients who are newly diabetics to prevent complications or detect them earlier.

The increased awareness of diabetic patients about DR will probably lead to a lower incidence of the advanced disease, especially with the developments in technology and artificial intelligence (AI) [21]. AI can be used as a screening or diagnostic tool. This is accomplished by computer exposure to databases; these can then be used to screen and diagnose. This is implemented in ophthalmology for diseases of a high incidence where an early diagnosis is crucial for treatment, such as in the case of DR, age-related macular degeneration (ARMD), glaucoma, retinopathy of prematurity (ROP), age-related or congenital cataract, and retinal vein occlusion (RVO) [22]. Recent studies suggested that AI can be a useful tool for diabetic retinopathy screening, prediction, and management [23,24,25,26,27]. In an era affected by the COVID-19 pandemic, the value of artificial intelligence and digital health technologies might lead to a more frequent use of AI in DR treatment and management [28]. The only hurdle which remains towards improving the screening and management of DR will become patient awareness and perception about this disease.

To the best of our knowledge, the practice and barriers regarding diabetic retinopathy were not well documented among diabetics [29,30].

## 5. Conclusions

There is a remarkable increase in the awareness of DR among the Saudi population. This might reflect the efforts made by physicians and healthcare authorities to achieve the goals of preventing the complications of DM and treating them earlier. Efforts to reach these goals might be aided by the new developments in screening and diagnosis tools for DR (such as AI). Achieving these goals will reduce the burdens on the healthcare system and improve the quality of life of the populations.

## Figures and Tables

**Table 1:** Bio-demographic data of diabetic patients, Tabuk region, Saudi Arabia.

Bio-Demographic Data	No	%
Age		
<35	66	17.1%
35–50	156	40.4%
>50	164	42.5%
Gender		
Male	183	47.4%
Female	203	52.6%
Education		
Non educated	51	13.2%
Basic education	69	17.9%
High school	119	30.8%
University/above	147	38.1%
Monthly income		
<5000 SR	161	41.7%
5000–10,000 SR	109	28.2%
11,000–20,000 SR	85	22.0%
>20,000 SR	31	8.0%
Smoking		
Yes	83	21.5%
No	303	78.5%
Co-morbidities		
None	141	36.5%
HTN	156	40.4%
Renal disease	29	7.5%
Cardiac diseases	61	15.8%
Hyperlipemia	111	28.8%
Rheumatological disease	45	11.7%

**Table 2:** Diabetes data and related eye diseases among patients, Tabuk region, Saudi Arabia.

Diabetes Data	No	%
Type of diabetes		
T1DM	131	33.9%
T2DM	188	48.7%
Don’t know	67	17.4%
Duration of DM (years)		
<5	112	29.0%
5–10	129	33.4%
>10	145	37.6%
The management of your diabetic		
Diet and exercise	79	20.5%
Oral hypoglycemic drug	202	52.3%
Insulin	172	44.6%
Frequency of check of DM at home		
When I feel unwell	119	30.8%
Once a month	33	8.5%
Once a week	53	13.7%
Once a day	88	22.8%
More than once a day	93	24.1%
Do you have eye disease?		
Yes	184	47.7%
No	202	52.3%
What are the diseases		
Cataract	31	16.8%
Diabetic retinopathy	33	17.9%
Unknown	16	8.7%
Glaucoma	17	9.2%
Refractive error	87	47.3%
Receive any treatment for eye disease?		
Yes	117	63.6%
No	67	36.4%

**Table 3:** Diabetic patients’ knowledge regarding diabetes and diabetic retinopathy, Tabuk, Saudi Arabia.

Knowledge Items		No	%
Do you know that a diabetic patient has to have an eye check-up regardless of blood sugar level?	Yes	283	73.3%
	No	11	2.8%
	Do not know	92	23.8%
How frequently should a diabetic patient undergo an eye check-up?	Only when vision affected	27	7.0%
	Every 2 Years	20	5.2%
	Yearly	146	37.8%
	Every 6 months	158	40.9%
	Do not know	35	9.1%
Diabetic patients need to have their eyes checked up only when they have symptoms.	Yes	148	38.3%
	No	196	50.8%
	Do not know	42	10.9%
Diabetes mellites can affect the retina.	Yes	310	80.3%
	No	22	5.7%
	Do not know	54	14.0%
The complications of diabetes are always symptomatic	Yes	216	56.0%
	No	79	20.5%
	Do not know	91	23.6%
Diabetes could be harmful to the eyes.	Yes	326	84.5%
	No	21	5.4%
	Do not know	39	10.1%
Diabetes can lead to blindness.	Yes	251	65.0%
	No	36	9.3%
	Do not know	99	25.6%
There are available treatments for Diabetic retinopathy.	Yes	103	26.7%
	No	40	10.4%
	Do not know	243	63.0%
You can prevent Diabetic eye complications by blood sugar control.	Yes	270	69.9%
	No	20	5.2%
	Do not know	96	24.9%
You can prevent Diabetic eye disease by regular visits to an ophthalmologist.	Yes	293	75.9%
	No	29	7.5%
	Do not know	64	16.6%
You can halt the progression of diabetic eye disease by good blood pressure control.	Yes	208	53.9%
	No	22	5.7%
	Do not know	156	40.4%
You can halt the progression of diabetic eye disease by good lipid control.	Yes	176	45.6%
	No	41	10.6%
	Do not know	169	43.8%

**Table 4:** Diabetic patients practice regarding eye check-up and screening, Tabuk region, Saudi Arabia.

Patient’s Practice		No	%
Have you had your eyes checked before?	Yes	282	73.1%
	No	104	26.9%
If yes, when was your last visit?	2 years ago	70	24.8%
	1 year ago	79	28.0%
	Less than 6 months ago	92	32.6%
	1 month ago	26	9.2%
	1 week ago	15	5.3%
How often do you go for an eye screening?	I did not visit the doctor	173	44.8%
	Monthly	32	8.3%
	Once every six months	83	21.5%
	Yearly	98	25.4%
If you did not have an eye screening in the past 12 months, what is the reason?	I did not receive any recommendations from the doctor	72	18.7%
	I did not have enough time	64	16.6%
	There is a difficulty to reach an ophthalmologist	38	9.8%
	I did not have symptoms or vision problems	36	9.3%
	I did not have the information about diabetic eye diseases	31	8.0%
	Too many other examinations and medical appointments	25	6.5%
	I did not have the information about retinal screening	23	6.0%
	I am afraid of the examination, result, or treatment	14	3.6%
	It is not necessary because my blood sugar is well controlled	13	3.4%
	I did not have money	11	2.8%
	I feel discomfort during the examination (eye drops, dilated pupils)	4	1.0%
What are the reasons that made you undergo your first eye screening?	Doctor’s referral	168	43.5%
	I know the importance of regular eye examination	60	15.5%
	I know the risk of diabetes in the retina	48	12.4%
What do you think was the biggest barrier for not getting an eye screening earlier?	Lack of knowledge	148	38.3%
	Lack of access to eye care	94	24.4%
	Time limitations	64	16.6%
	Fear of discovering something bad	46	11.9%
	Cost/insurance issue	34	8.8%

**Table 5:** Patients attitude regarding physician’s role and eye screening, Tabuk region, Saudi Arabia.

Physician Role and Patient Attitude	No	%
Did your primary physician mention that you need a regular follow-up for DR?		
Yes	223	57.8%
No	163	42.2%
Have you been informed by the primary physician about the effect of DM on your eyes?		
Yes	224	58.0%
No	162	42.0%
Were you advised by your primary physician to go to an ophthalmologist for eye screening?		
Yes	225	58.3%
No	161	41.7%
Were you formally referred by your primary physician for eye screening?		
Yes	200	51.8%
No	186	48.2%
Do you think that a diabetic patient needs to have an eye check-up when his/her blood sugar level is well-controlled?		
Agree	279	72.3%
Disagree	28	7.3%
Do not know	79	20.5%
Do you think that a diabetic patient needs to have an eye check-up when his/her blood sugar level is poorly controlled?		
Agree	262	67.9%
Disagree	33	8.5%
Do not know	91	23.6%

**Table 6:** Determinants of patients’ knowledge level regarding diabetes and diabetic retinopathy among diabetic patients, Tabuk region, Saudi Arabia.

Factors		Knowledge Level				*p*-Value
		Poor		Good		
		No	%	No	%	
Age	<35	39	59.1%	27	40.9%	0.030 *
	35–50	62	39.7%	94	60.3%	
	>50	74	45.1%	90	54.9%	
Gender	Male	75	41.0%	108	59.0%	0.103
	Female	100	49.3%	103	50.7%	
Education	Non educated	34	66.7%	17	33.3%	0.006 *
	Basic education	33	47.8%	36	52.2%	
	High school	51	42.9%	68	57.1%	
	University/above	57	38.8%	90	61.2%	
Duration of DM (years)	<5	69	61.6%	43	38.4%	0.001 *
	5–10	60	46.5%	69	53.5%	
	>10	46	31.7%	99	68.3%	
Type of diabetes	T1DM	40	30.5%	91	69.5%	0.001 *
	T2DM	93	49.5%	95	50.5%	
	Do not know	42	62.7%	25	37.3%	
Frequency of checking the blood sugar at home	When I feel unwell	59	49.6%	60	50.4%	0.732
	Once a month	15	45.5%	18	54.5%	
	Once a week	24	45.3%	29	54.7%	
	Once a day	40	45.5%	48	54.5%	
	More than once a day	37	39.8%	56	60.2%	
Do you have any eye diseases?	Yes	82	44.6%	102	55.4%	0.771
	No	93	46.0%	109	54.0%	
The most common Source for your information about DR	None	63	69.2%	28	30.8%	0.001 *
	Family and friends	43	46.2%	50	53.8%	
	Internet & social media	26	36.6%	45	63.4%	
	Doctors	43	32.8%	88	67.2%	
Have you been informed by the primary physician about the effect of DM on your eyes	Yes	72	32.1%	152	67.9%	0.001 *
	No	103	63.6%	59	36.4%	
Did you have your eyes checked up before?	Yes	106	37.6%	176	62.4%	0.001 *
	No	69	66.3%	35	33.7%	

*p*: Pearson X^2^ test; * *p* < 0.05 (significant).

## Data Availability

All the data presented in this manuscript are available on request.
